# Correlation of cardiac function and cerebral perfusion in a murine model of subarachnoid hemorrhage

**DOI:** 10.1038/s41598-021-82583-9

**Published:** 2021-02-08

**Authors:** Axel Neulen, Michael Molitor, Michael Kosterhon, Tobias Pantel, Elisa Holzbach, Wolf-Stephan Rudi, Susanne H. Karbach, Philip Wenzel, Florian Ringel, Serge C. Thal

**Affiliations:** 1 Department of Neurosurgery, University Medical Center of the Johannes Gutenberg–University Mainz, Langenbeckstrasse 1, 55131 Mainz, Germany; 2 Center for Cardiology–Cardiology I, University Medical Center of the Johannes Gutenberg–University Mainz, Mainz, Germany; 3 Center for Thrombosis and Hemostasis (CTH), University Medical Center of the Johannes Gutenberg–University Mainz, Mainz, Germany; 4 German Center for Cardiovascular Research (DZHK)-Partner Site Rhine-Main, Mainz, Germany; 5 Department of Anesthesiology, University Medical Center of the Johannes Gutenberg–University Mainz, Langenbeckstrasse 1, 55131 Mainz, Germany; 6 Center for Molecular Surgical Research (MFO), University Medical Center of the Johannes Gutenberg–University Mainz, Mainz, Germany

**Keywords:** Cardiology, Neurology

## Abstract

Cerebral hypoperfusion is a key factor for determining the outcome after subarachnoid hemorrhage (SAH). A subset of SAH patients develop neurogenic stress cardiomyopathy (NSC), but it is unclear to what extent cerebral hypoperfusion is influenced by cardiac dysfunction after SAH. The aims of this study were to examine the association between cardiac function and cerebral perfusion in a murine model of SAH and to identify electrocardiographic and echocardiographic signs indicative of NSC. We quantified cortical perfusion by laser SPECKLE contrast imaging, and myocardial function by serial high-frequency ultrasound imaging, for up to 7 days after experimental SAH induction in mice by endovascular filament perforation. Cortical perfusion decreased significantly whereas cardiac output and left ventricular ejection fraction increased significantly shortly post-SAH. Transient pathological ECG and echocardiographic abnormalities, indicating NSC (right bundle branch block, reduced left ventricular contractility), were observed up to 3 h post-SAH in a subset of model animals. Cerebral perfusion improved over time after SAH and correlated significantly with left ventricular end-diastolic volume at 3, 24, and 72 h. The murine SAH model is appropriate to experimentally investigate NSC. We conclude that in addition to cerebrovascular dysfunction, cardiac dysfunction may significantly influence cerebral perfusion, with LVEDV presenting a potential parameter for risk stratification.

## Introduction

Spontaneous subarachnoid hemorrhage (SAH), a type of hemorrhagic stroke, is caused primarily by rupture of an intracranial aneurysm^1,2^. There are two major phases of brain injury after SAH, early and delayed, with both shared and distinct causative factors. Early brain injury (EBI) occurs as an immediate result of bleeding and associated transient global cerebral ischemia^1,3^. Alternatively, delayed cerebral ischemia (DCI), occurring mostly within 21 days after SAH onset, demonstrates a multifactorial pathogenesis, including vasospasms of microvessels and macrovessels, microthrombosis, and cortical spreading depressions^3,4^. Clinically, DCI manifests as new neurological deficits related to cerebral ischemia^5^. Cerebral hypoperfusion is central to the development of both EBI and DCI^3^ and, therefore, may be a promising target for therapeutic intervention to improve the neurological outcome.

SAH impairs cardiac function by disrupting the neuro-cardiac axis, leading to neurogenic stress cardiomyopathy (NSC) of variable severity [reviewed in^6,7^]. Under physiological conditions, cerebral autoregulation attenuates the effects of cardiac function on cerebral perfusion. However, cerebral autoregulation is disrupted after SAH^5,8^. Further, several studies have shown that decreased cardiac output (CO) can induce both acute^9,10^ and chronic cerebral hypoperfusion^11,12^, and that impaired left ventricular (LV) function is associated with cognitive decline^13,14^. Presumably, impaired LV function leads to cerebral hypoperfusion after SAH. This assumption is supported by a clinical study that found an association between cardiac dysfunction and cerebral hypoperfusion during the first 24 h after hospital admission in patients with SAH^15^. Based on these clinical findings^5–15^, improving cardiac function appears essential for mitigating cerebral hypoperfusion and optimal neurological outcome after SAH. Nevertheless, this approach has never been studied. Rather, current treatment guidelines focus on the importance of cerebral perfusion pressure (CPP) and the role of the cerebral vasculature in cerebral perfusion^16–19^.

Murine models are an increasingly important research tool to study SAH pathophysiology. Similar to the human disease, rodents develop cerebral hypoperfusion after experimental induction of SAH^20–22^. Histological studies have reported myocardial injury 7 days after SAH induction in mice^23–25^, suggesting that murine models are suitable for studies on the neuro-cardiac axis. Cerebral hypoperfusion after SAH is pivotal to both EBI and DCI pathophysiology^26–28^, so most experimental studies have focused on the cerebral vasculature’s role in dysregulating cerebral circulation^29–32^. However, the link between post-stroke cardiac dysfunction and cerebral hypoperfusion has also not been established in experimental studies.

Therefore, we investigated the association between cerebral perfusion and cardiac function in a murine endovascular filament perforation model of SAH. The primary purpose was to analyze the correlation between cardiac function and cerebral cortical perfusion after SAH induction. We also examined electrocardiographic (ECG) and echocardiographic signs indicative of NSC to facilitate future interventional studies for improving CO and cerebral perfusion following SAH.

## Methods

### Ethics approval

The animal experiments were approved by the responsible animal care committee (Landesuntersuchungsamt Rheinland-Pfalz) and conducted in accordance with the German Animal Welfare Act (TierSchG). All applicable international, national, and institutional guidelines for the care and use of animals were followed. The study was carried out in compliance with the ARRIVE guidelines.

### Animals and housing conditions

Female C57BL/6N mice (Janvier, Saint-Berthevin Cedex, France; aged 11–12 weeks) were used for all experiments. As described in our previous study^22^, the mice were housed under controlled environmental conditions (12-h light–dark cycles, 23 ± 1 °C, 55% ± 5% relative humidity) with free access to water and food (Altromin, Lage, Germany). Body weight was measured daily during the experimental duration.

### Study design

**Figure 1:**
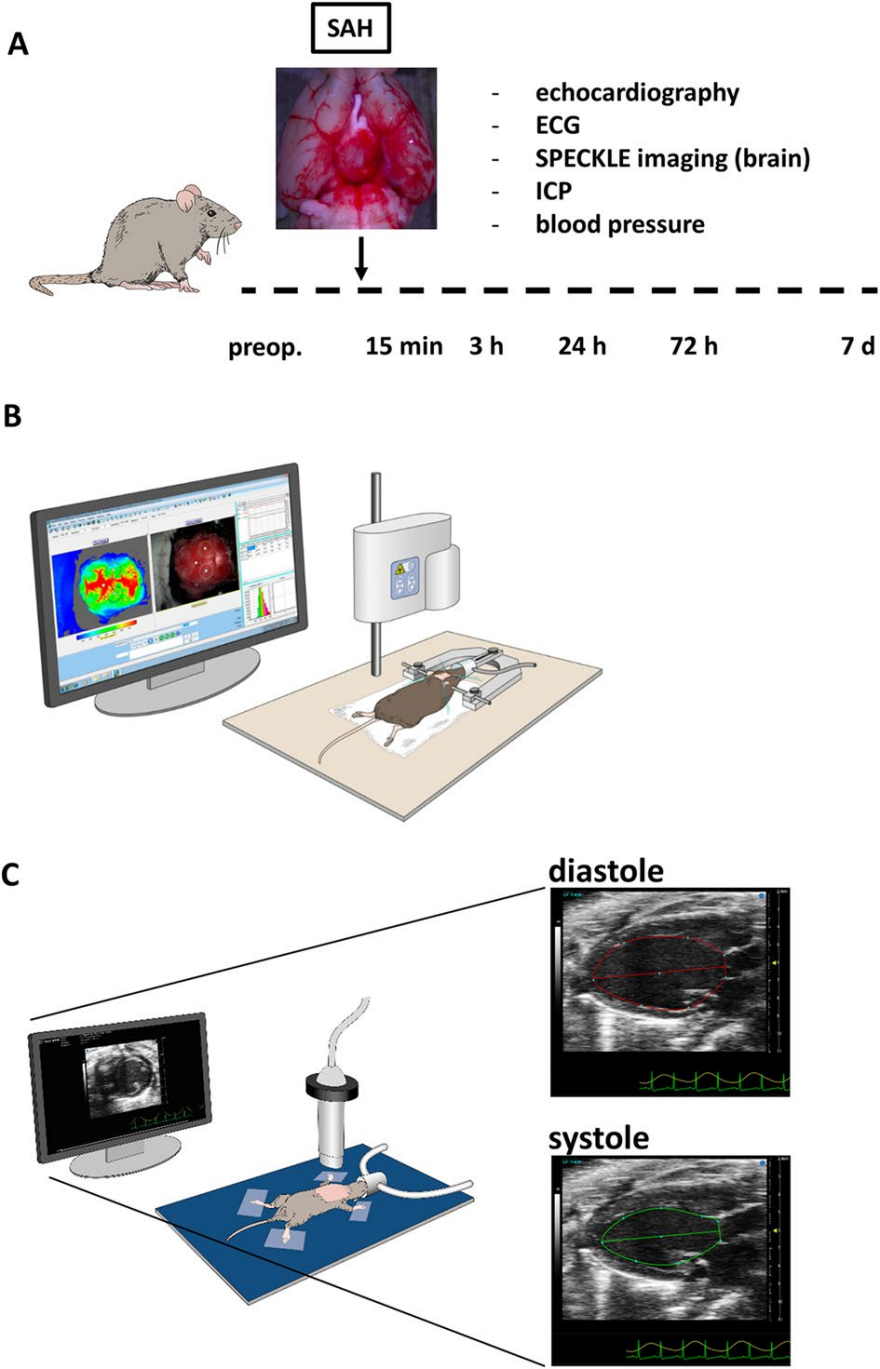
Study concept. (**A**) Study design. Mice were randomized to sham surgery and experimental subarachnoid hemorrhage (SAH) groups and subjected to serial measures of cerebral perfusion and cardiac function at the indicated times. (**B**) Schematic illustration of the SPECKLE imaging method for measuring cerebral cortical perfusion. (**C**) Illustration of high-frequency small animal transthoracic echocardiography for assessing cardiac output (CO), stroke volume, left ventricular ejection fraction (LVEF), and left ventricular end-diastolic volume (LVEDV).

Twenty mice were randomized to receive either SAH (13 animals) or sham surgery (7 animals). Cerebral cortical blood flow and intracranial pressure (ICP) were determined before surgery and 15 min, 3 h, 24 h, 72 h, and 7 days after SAH induction or sham surgery. ECG and echocardiography were performed at baseline on the day before surgery, and immediately after each post-surgical cerebral cortical blood flow measurement. Mice were sacrificed after the last echocardiography exam, 7 days post-surgery. Non-invasive measurements of arterial blood pressure were acquired on the day before surgery, and on days 1, 3, and 7 after surgery, using the Coda mouse tail-cuff system (Kent-Scientific, Torrington, CT, USA). The study design is shown in Fig. 1.

### Murine model of SAH

SAH was induced by endovascular filament perforation, under isoflurane anesthesia (Abbvie, Wiesbaden, Germany) with continuous ICP monitoring^21,22,32,33^. Anesthesia was induced with 4% isoflurane for 1 min and maintained with 2% isoflurane. For analgesia, Buprenorphin (Indivior, Slough, Berkshire, UK; 0.1 mg/kg body weight) was subcutaneously injected before surgery and then three times per day until the end of the experiment on post-surgical day 7. Body temperature was maintained at 36 °C during surgery using a heating pad (Physiotemp Instruments LLC, Clifton, NJ, USA).

For SAH induction, the anaesthetized mice were first placed in the prone position, and an ICP probe (Codman, Johnson & Johnson, Raynham, MA, USA) was placed in the right frontal region through a burr hole. After surgical preparation, a filament (Prolene 5.0; Ethicon, Norderstedt, Germany) was inserted into the left external carotid artery and intracranially advanced through the internal carotid artery^21,22,32,33^. A sharp rise in ICP was considered as an indicator of successful endovascular perforation. The ICP probe was removed at the end of the procedure. Surgical wounds were closed using Prolene 6.0 sutures (Ethicon). The same procedure was conducted in the sham group, but without advancing the endovascular filament into the internal carotid artery. After surgery, the animals were kept in an incubator, heated to 36 °C (IC8000; Draeger, Luebeck, Germany) for 1 h, to prevent hypothermia.

### Determination of cerebral cortical perfusion with laser SPECKLE contrast imaging

All measurements were performed under anesthesia (2% isoflurane) while maintaining the body temperature at 36 °C using a heating pad (Physitemp Instruments LLC). Cerebral cortical perfusion was determined, using a laser perfusion imager (MoorFLPI-2-blood flow imager; Cologne, Germany), and analyzed with Moor review software (moorFLPI Full-Field Laser Perfusion Imager Review Version 4.0) as described^21,22^. Briefly, following a midline incision to expose the calvaria, 60 transcalvarian perfusion images were recorded at one picture per second. The wound was then closed with Prolene 6.0 sutures. A mean image was calculated from the 60 perfusion images. The mean flux values were determined by evaluating a 3-mm diameter region of interest (ROI) over the perfusion territory of the left middle cerebral artery. Changes in perfusion are expressed relative to the preoperative flux value. This method is illustrated in Fig. 1.

### High-frequency small animal echocardiography and ECG recording

Cardiac function was assessed by transthoracic echocardiography. Serial examinations were performed under isoflurane anesthesia (1.2–1.5%) using the Vevo3100 high-resolution imaging system (VisualSonics, FujiFilm, Toronto, Canada) equipped with a 38 MHz (MZ 400) linear array transducer. Images were acquired at above 200 frames/s. The one-lead ECG and breathing rate were monitored throughout, and the mouse’s body temperature was kept stable during anesthesia using a heating system within the handling platform. Brightness (B)-mode and Motion (M)-mode movies of the parasternal long axis (PLAX) and parasternal short axis (SAX, mid ventricular) were acquired. Post-acquisition analysis was performed using VevoLab Software (VisualSonics). Left ventricular ejection fraction (LVEF), left ventricular end-diastolic volume (LVEDV), CO, and stroke volume were measured as LV function indices.

### Statistical analysis

GraphPad Prism software (La Jolla, CA, USA) was used for correlation analyses, and SigmaPlot version 12.5 (Systat Software, San Jose, CA, USA) was used for descriptive statistics and statistical power analyses. Data are presented as mean ± standard error of the mean (SEM). The data were tested for normal distribution using the Kolmogorow-Smirnow normality test. To compare variables between 2 groups, group means (sham vs. SAH) were compared by Mann–Whitney *U* test in case of non-normal distribution and Student’s t-test in case of normal distribution. A p < 0.05 (two-tailed) was considered statistically significant. Association strengths were measured by Pearson’s correlation coefficients in case of normal distribution and by Spearman’s correlation coefficients in case of non-normal distribution, again with a p < 0.05 considered significant.

Group sizes were calculated based on the mortality rates in our previous experimental SAH studies^21,22^ and statistical power analysis with alpha of 0.05, power of 0.8, and assuming an r value of 0.85.

## Results

### Mortality and body weight

13 animals were randomized to SAH induction and seven to sham surgery. Two SAH animals and one sham animal died within 3 h after surgery, and one SAH animal died within 24 h after surgery. These animals were excluded from the evaluation. Of the remaining 10 SAH animals, one showed subdural hematoma over the left hemisphere, which precluded measuring cerebral cortical perfusion. Therefore, this animal was also excluded from the evaluation, leaving nine SAH and six sham animals. One further SAH animal was found dead on day 3 before the perfusion measurement. Another three SAH animals and three sham animals died on day 4 after surgery. Accordingly, statistical analyses were performed on 9 SAH group animals for the time points until 24 h post-surgery, 8 animals for the 72 h time point, and on 5 animals at 7 days post-SAH. In the sham group, statistical analyses were performed on 6 animals for the time points until 72 h post-surgery and on 3 animals at 7 days post-surgery. In the sham group, cerebral perfusion in 1 animal at 7 days and in 2 animals at 72 h post-surgery, and the cardiac parameters in 1 animal at the time points before surgery and 15 min post-surgery could not be evaluated due to technical reasons.

The body weights were similar between SAH and sham animals at all measurement points post-surgery but differed slightly (~ 5%) at baseline (before surgery: 20.0 ± 0.5 g vs. 18.9 ± 0.1 g, p < 0.05; day 1: 18.5 ± 0.3 vs. 18.1 ± 0.4 g; day 3: 17.1 ± 0.7 vs. 15.8 ± 0.5 g; day 7: 19.1 ± 0.4 vs. 19.0 ± 1.0 g).

### Elevated ICP and cerebral hypoperfusion after SAH

Mice were randomized to either SAH induction or sham surgery. Cerebral perfusion was determined along with ICP, ECG, and echocardiography at baseline and 15 min, 3 h, 24 h, 72 h, and 7 days after SAH or sham surgery. The study protocol is illustrated in Fig. 1.

**Figure 2:**
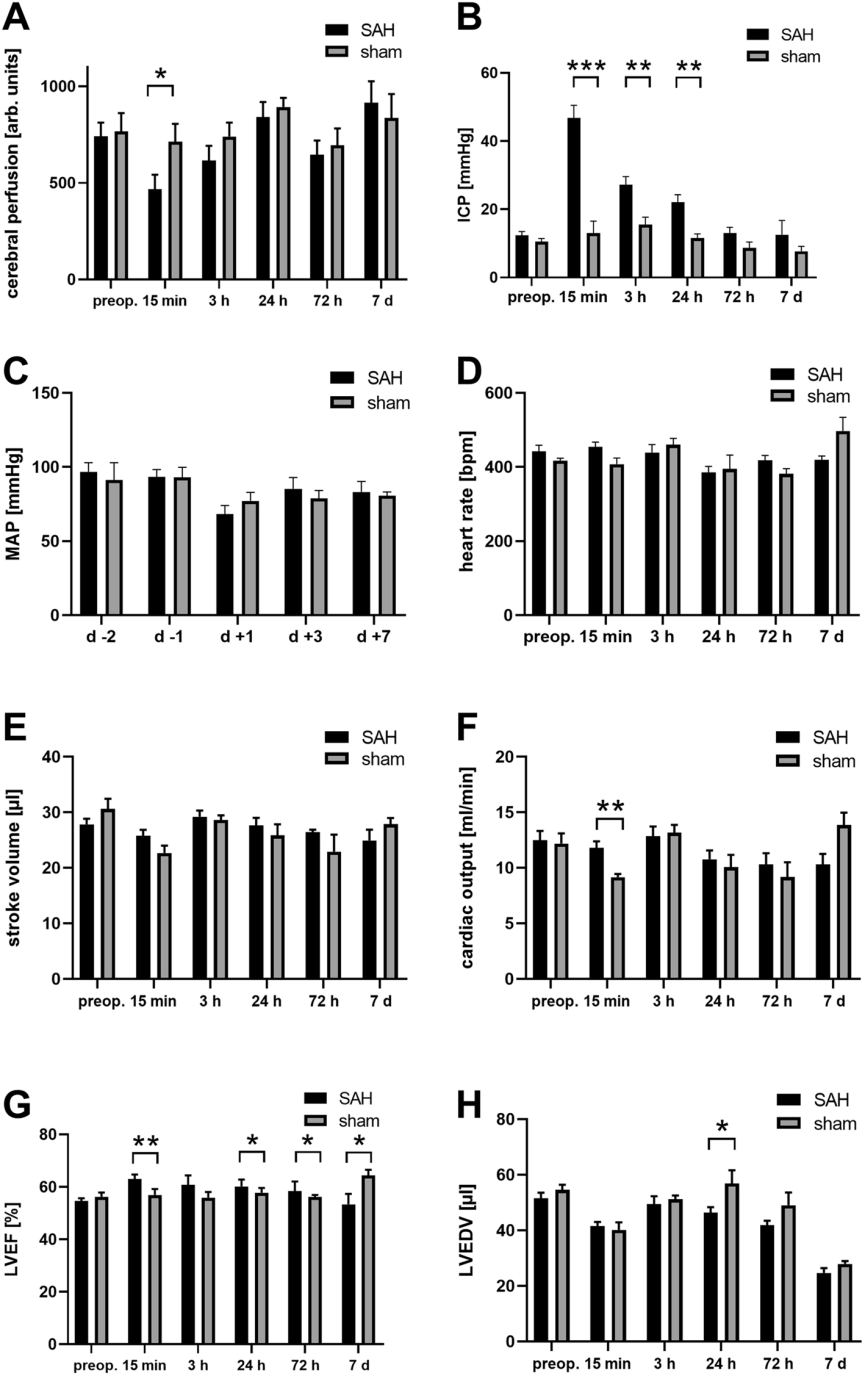
Comparisons of cerebral and cardiovascular parameters between SAH and sham groups. (**A**)–(**H**) Cerebral and cardiovascular parameters were measured from SAH and sham animals before surgery and 15 min, 3 h, 24 h, 72 h, and 7 days post-surgery. SAH: n = 9 for the time points before surgery and 15 min, 3 h, 24 h postop.; n = 8 at 72 h postop.; n = 5 at 7 days postop.; Sham: cerebral perfusion: n = 4 at 72 h and n = 2 at 7 days post-surgery; heart reate, stroke volume, cardiac output, LVEF, LVEDV: n = 5 for the time points before surgery and 15 min post-surgery; in all other cases: n = 6 for the time points before surgery and 15 min, 3 h, 24 h, and 72 h post-surgery; n = 3 for 7 days post-surgery. Data are presented as mean ± SEM; *p < 0.05, **p < 0.01, ***p < 0.001 (Mann–Whitney U test or Student's t-test as appropriate). arb: arbitrary; ICP: intracranial pressure; LVEDV: left ventricular end-diastolic volume; LVEF: left ventricular ejection fraction; MAP: mean arterial pressure; SAH: subarachnoid hemorrhage; SEM: standard error of the mean.

We first examined whether SAH induces a reduction in cerebral perfusion. Similar to previous reports^20–22,29–31^, SAH induction led to a rapid and significant increase in ICP after endovascular perforation (SAH: 71.3 ± 9.7, sham 11.3 ± 2.5 mmHg, p < 0.001), which normalized slowly. Induction of SAH also caused a significant decrease in cortical perfusion, which recovered to near baseline by 24 h, again consistent with previous studies^20–22,29–31^. Mean arterial pressure (MAP) decreased after surgery, but the difference between SAH and sham animals did not reach statistical significance. The detailed time courses of these physiological parameters are shown in the Fig. 2.

### Pathophysiological features of NSC after SAH

**Figure 3:**
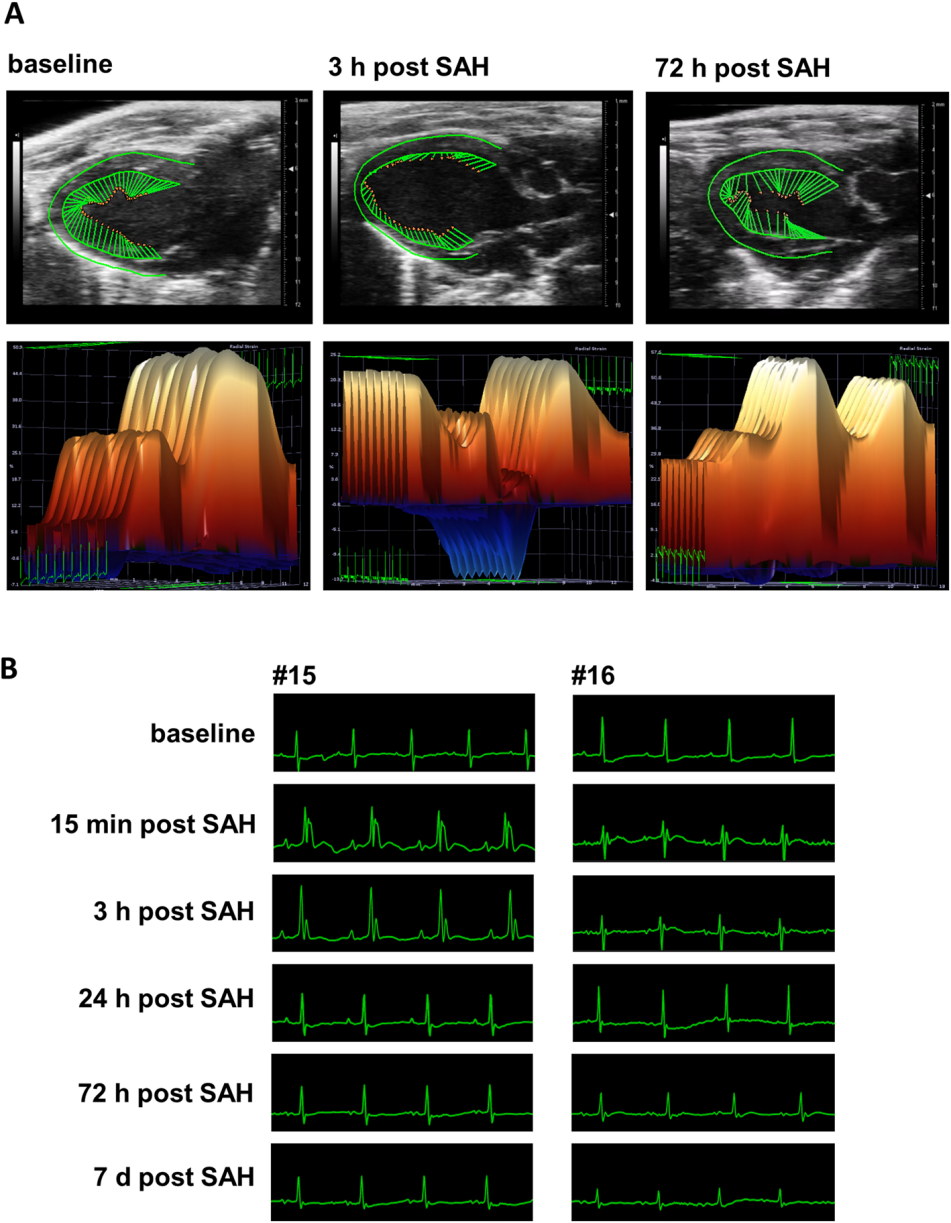
ECG and echocardiographic features of neurogenic stress cardiomyopathy (NSC) in mice subjected to experimental subarachnoid hemorrhage (SAH). (**A**) Echocardiographic findings from one of two SAH group animals in which the left ventricular function was markedly reduced at 3 h post-surgery (in contrast to the overall changes in the SAH group but consistent with emergence of NSC in a subset of SAH patients). Recovery was complete by 72 h post-surgery. A video of the echocardiography can be found in the supplementary material. (**B**) An example of abnormal electrocardiographic findings (right bundle branch block) observed in three mice after SAH induction. Recovery was complete at 24 h post-SAH.

We then investigated whether SAH animals showed echocardiographic or electrocardiographic signs of NSC. We observed abnormal echocardiographic or electrocardiographic changes in only a subset of SAH model mice, in accord with clinical observations^6,7,34,35^. In two of ten SAH group animals, LVEF was reduced substantially at 3 h post-SAH despite an overall increase in LVEF after SAH compared to sham (Fig. 3 and supplementary material (video)). Three of ten SAH group animals exhibited a right bundle branch block at 15 min and 3 h post-surgery. In one animal, this conduction block was clearly associated with a wall-motion abnormality. None of these pathological findings were present at baseline, confirming SAH-dependent induction, and all abnormal ECG or echocardiographic findings recovered by 24 h post-surgery.

We also investigated whether there was an overall reduction in LV function among the SAH group animals compared to the sham controls. In the SAH group, stroke volume and LVEF were elevated for up to 72 h post-surgery, with a significant group difference at 15 min, 24 h, and 72 h for LVEF, while CO was elevated at 15 min, 24 h, and 72 h, with a significant group difference at 15 min. Conversely, LVEF was significantly higher in sham animals at 7 days post-surgery. Similarly, LVEDV was higher in the sham group at 24 h and 72 h post-surgery, which reached the level of significance at 24 h.

Taken together, our data show overall enhanced LV function until 72 h post-surgery and reduced cardiac contractility at 7 days post-surgery in the SAH group compared to sham animals (Fig. 2). However, a few animals showed impaired LV systolic function resembling severe human NSC at 15 min and 3 h post-SAH.

### Cardiac function correlates with cerebral perfusion after SAH

**Figure 4:**
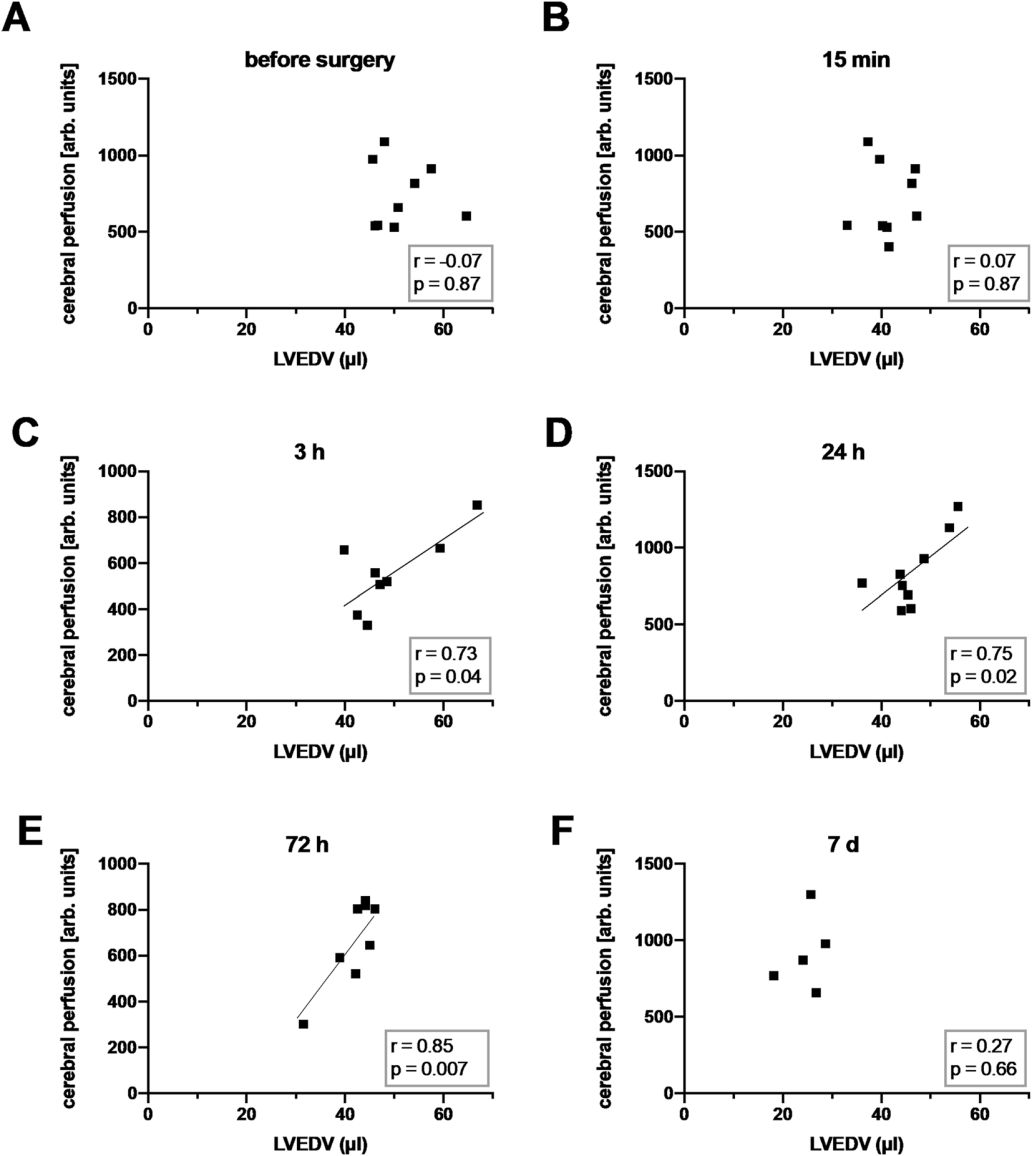
Correlations of LVEDV with cerebral perfusion after SAH. (**A**)–(**F**) Correlations of left ventricular end-diastolic volume (LVEDV) with cerebral perfusion at the indicated times post-surgery. Extended analyses can be found in the supplementary material. n = 9 for the time points before surgery and 15 min, 3 h, 24 h postop.; n = 8 at 72 h postop.; n = 5 at 7 days postop. Correlation is expressed by Pearson’s or Spearman’s correlation coefficient (r) as appropriate, with p < 0.05 considered statistically significant.

**Figure 5:**
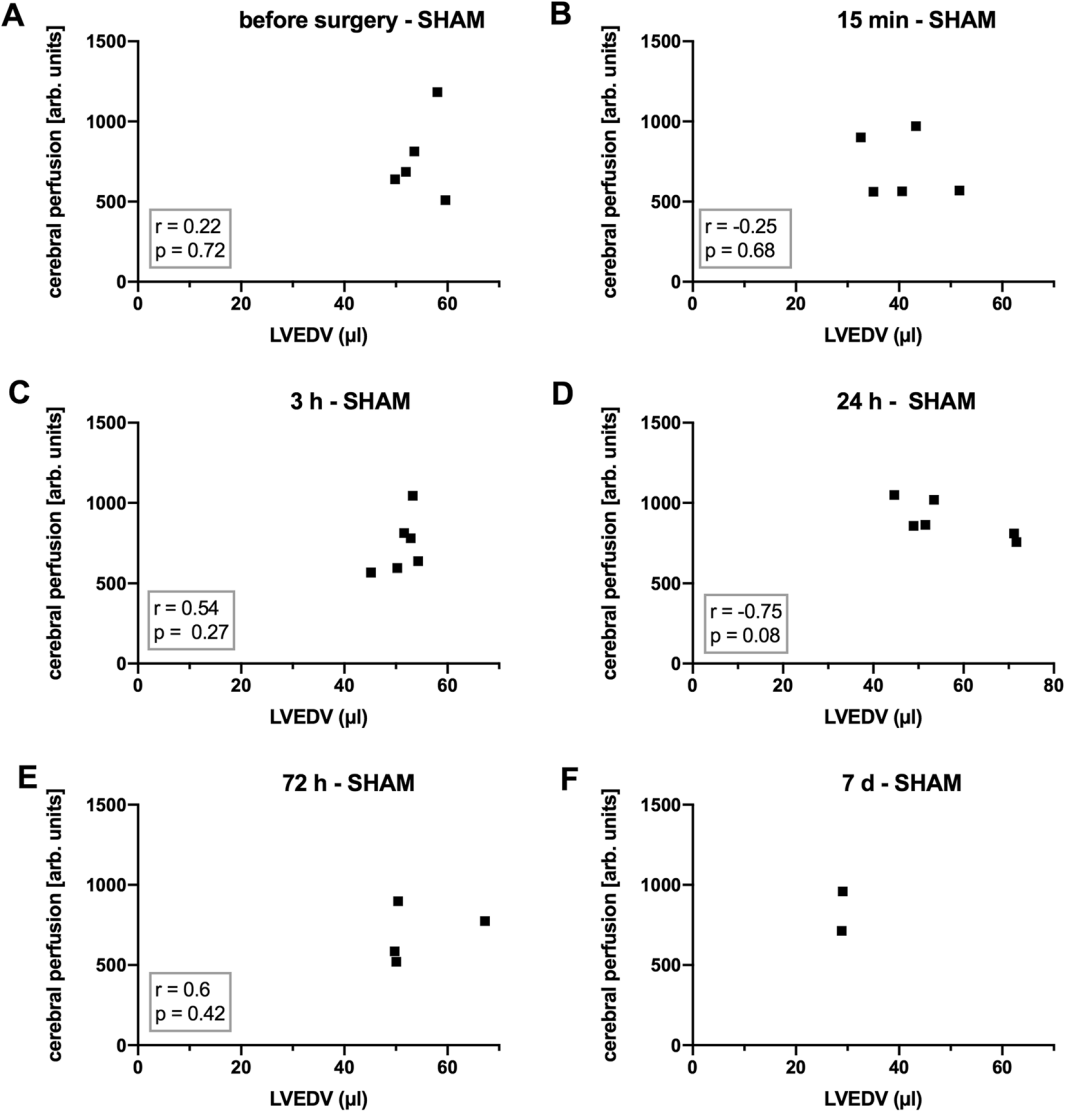
Correlations of LVEDV with cerebral perfusion in sham animals. (**A**)–(**E**) Correlations of left ventricular end-diastolic volume (LVEDV) with cerebral perfusion at the indicated times post-surgery. (**F**) For 7 days postop., a correlation analysis was not performed due to the case number. n = 5 for the time points before surgery and 15 min post-surgery; n = 6 for 3 h and 24 h postop.; n = 4 at 72 h and n = 2 at 7 days postop. Correlation is expressed by Pearson’s or Spearman’s correlation coefficient (r) as appropriate, with p < 0.05 considered statistically significant.

Cardiac output reportedly influences cerebral perfusion and function^9–11,13^, so we next tested whether LV function impacts post-SAH cerebral perfusion. To this end, we examined the correlations of cardiac parameters, determined by high-frequency ultrasound, with cerebral cortical perfusion determined by SPECKLE imaging. LVEDV correlated significantly with cerebral perfusion at 3 h, 24 h, and 72 h post-SAH, which was not the case in sham animals. This indicates a link between diastolic filling and cerebral perfusion at these time points. There was a trend for correlation of cardiac stroke volume and CO with cerebral perfusion at 72 h after cranial bleeding, which, however, did not reach statistical significance. This reveals a link between systolic left ventricular output and cerebral perfusion at this time point. Alternatively, there was no significant correlation between cerebral perfusion and LVEF (Figs. 4, 5, and supplementary material (Figs. E1–E4)).

## Discussion

To the best of our knowledge, this is the first study to analyze the relationship between cardiac function and cerebral perfusion in a murine model of SAH. The most important findings of our study are: (i) Overall, SAH enhances LV contractility in the early period after SAH but with marked inter-individual variability, (ii) only a specific subset of animals developed echocardiographic and ECG signs of NSC, a pattern similar to SAH patients^6,7,34,35^, and (iii) LVEDV correlated significantly with cerebral perfusion at defined time points post-SAH, and there was a non-significant trend for correlation of cardiac output and stroke volume with cerebral perfusion. Taken together, these data show a relation between cerebral perfusion and cardiac function and therefore indicate that, in addition to cerebrovascular dysfunction, cardiac dysfunction may significantly influence cerebral perfusion, which presumably affects neurological outcome after SAH.

A substantial minority of SAH patients develop NSC, and post-SAH alterations in cardiac function have been linked to NSC development^6,7,34–36^. NSC symptoms range from shortness of breath to pulmonary edema, life-threatening cardiac arrhythmias, and congestive heart failure, and may be paralleled by increased biomarkers of myocardial injury, greater filling pressures, and abnormal ECG findings^6,7^. In a prospective clinical study, reduced LV function was observed in 15% and regional wall-motion abnormalities (RWMAs) without reduced LV function in 13% of 173 SAH patients in the first days after cranial bleeding^34^. Another prospective study reported pathological echocardiography findings in 17% of 63 SAH patients^35^. Neurogenic stress cardiomyopathy most likely results from excessive catecholamine release induced by the SAH, which leads to myocardial sympathetic overstimulation, excessive myocardial contraction, and ATP consumption, ensuing cardiomyocyte death, and finally cardiac inflammation^6,7,24,37–40^. NSC development has been linked to DCI and worse neurological outcome^6,7,35,36^.

Recent clinical research indicates that the degree of cerebral hypoperfusion during the first hours after SAH determines EBI severity, DCI occurrence, and neurological outcome^26–28^. Based on our experimental data, we suggest that cardiac dysfunction contributes directly to cerebral hypoperfusion and cerebral injury after SAH. This is supported by a clinical study that found an association between cardiac dysfunction and cerebral hypoperfusion during the first 24 h after hospital admission in SAH patients^15^. The similar patterns of cardiovascular and cerebrovascular dysfunction following both experimental SAH and clinical cases further validate this notion and suggest that our murine SAH model is well suited to study NSC and cardiac pathogenesis after SAH as well as to investigate novel therapeutic approaches.

We found a significant positive correlation between LVEDV and cerebral perfusion between 3 and 72 h post-SAH, as well as markedly lower LVEDV among individual animals. One possible reason for low LVEDV in some individuals (those showing the most severe hypoperfusion) could be a lack of intravascular volume, resulting in low preload and afterload. However, there were no obvious differences in body weight loss or intraoperative blood loss between animals with low and high LVEDV, making it unlikely that hypovolemia can account for poor post-SAH LVEDV. A clinical multicenter observational study, using pulse contour analysis, found that the global end-diastolic volume index (GEDI) was significantly lower on the 2 days preceding DCI in a group of SAH patients^41^. However, this apparent lack of intravascular volume was not caused by differences in fluid balance compared to patients without DCI. The strong correlation between LVEDV and cerebral perfusion at multiple early time points suggest that reduction in GEDI may directly contribute to DCI.

Another potential reason for reduced LVEDV is diastolic dysfunction. A clinical study observed diastolic dysfunction in 71% of 223 SAH patients, and diastolic dysfunction was associated with pulmonary edema^42^. However, a link between diastolic dysfunction and cerebral hypoperfusion had not been investigated. Therefore, we assessed the E / A relation, but found no difference between animals showing low or high LVEDV at time points with significant correlations between LVEDV and cerebral perfusion (3, 24, and 72 h post-SAH). Furthermore, there was no significant correlation between E/A and cerebral perfusion among SAH group animals or a significant difference in E/A between SAH and sham groups (supplementary material, Figs. E5, E6). These findings would argue against diastolic dysfunction accounting for low LVEDV and associated cerebral hypoperfusion; however, considering the sample sizes of our experimental study we cannot eliminate a contribution from diastolic dysfunction.

Finally, we want to address the limitations of our study. Firstly, only 9 SAH and 6 sham (control) animals entered the final analysis. Group sizes further decreased over time leaving 5 animals in the SAH group and 3 animals in the control group on day 7. Although statistical analysis revealed significant results, the small sample sizes need to be pointed out as a limitation of this study. Furthermore—because the measurements of perfusion and cardiac function were only performed at defined timepoints and not continuously—we cannot exclude that the lack of significance in several measures at the later time points is because the most affected mice were the ones that died. Secondly, a correlation between parameters of cardiac function and cerebral perfusion does not prove a causal relationship. We can furthermore not rule out that cerebral perfusion has an impact on cardiac function rather than cardiac function on cerebral perfusion. Nevertheless, our study demonstrates that a rodent model and a new experimental setting is available to experimentally investigate NCS and the relation of cardiac function and cerebral perfusion, and to potentially develop new therapeutic strategies to either prevent or treat NSC after SAH. Thirdly, the animals underwent repeated surgeries for imaging of cortical perfusion and ICP. Although the surgical trauma by the single measures was low and anesthesia times were short, stress imposed by the repeated surgeries as well as pharmacological effects of the anesthetic agents may present confounders. It should be noted, however, that the longitudinal nature of the study, which is made possible by the experimental setting used here, is a strength.

In summary, we show, to the best of our knowledge for the first time, that cerebral cortical hypoperfusion correlates with cardiac function after experimental SAH in mice. We conclude that in addition to cerebrovascular dysfunction, cardiac dysfunction may significantly influence cerebral perfusion, with LVEDV presenting a potential parameter for risk stratification. This finding may be of substantial clinical significance since it implies that both mitigation of cardiac dysfunction and suppression of cerebrovascular abnormalities, such as local cortical micro-vasospasm, are required to restore cerebral perfusion, which presumably is critical to obtain optimal neurological outcome. Our study confirms clinical approaches focusing on monitoring and optimizing cardiac function in patients suffering from SAH.

## Supplementary Information

Supplementary Information 1.

Supplementary Video 1.

## Data Availability

The data that support the findings of this study are available from the corresponding authors upon reasonable request.
