# Supplementary material for: Correlation of cardiac function and cerebral perfusion in a murine model of subarachnoid hemorrhage
Source: Sci Rep. 2021 Feb 8;11:3317. doi: 10.1038/s41598-021-82583-9 (PMC7870815; doi:10.1038/s41598-021-82583-9)
Supplement: Supplementary file 1 — Supplementary Information 1. [file 41598_2021_82583_MOESM1_ESM.pdf]

Correlation of cardiac function and cerebral perfusion in a murine model of subarachnoid hemorrhage

Axel Neulen, Michael Molitor, Michael Kosterhon, Tobias Pantel,

Elisa Holzbach, Wolf-Stephan Rudi, Susanne H. Karbach,

Philip Wenzel, Florian Ringel, Serge C. Thal

SUPPLEMENTARY MATERIAL

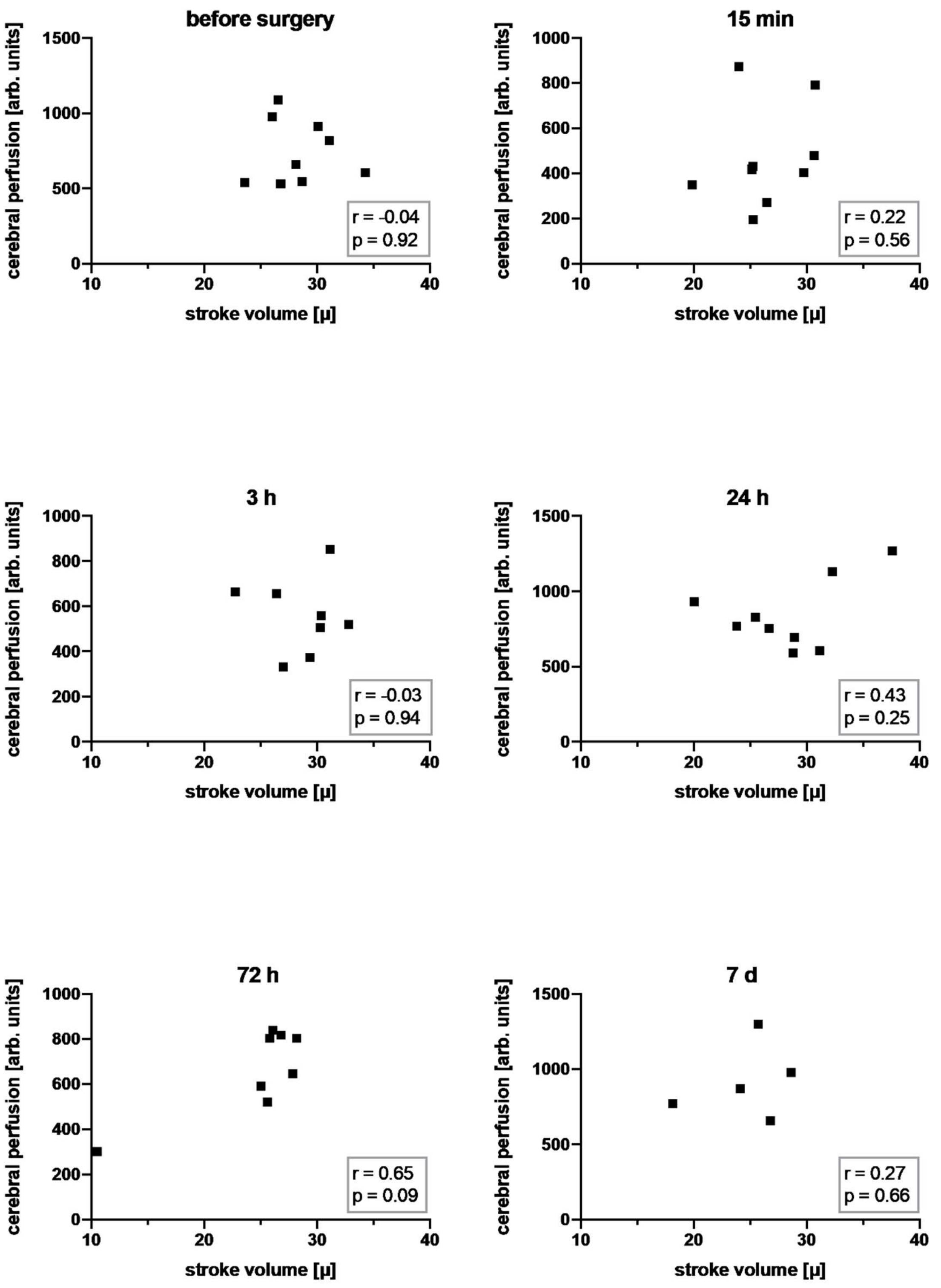

Supplemental Figure E1

Correlation of stroke volume with cerebral perfusion at the indicated times

Correlation strength, expressed as Pearson's or Spearman's correlation coefficient (r) as appropriate, with $p < 0.05$ considered statistically significant.

n = 9 for the time points before surgery and 15 min, 3 h, 24 h postop.; n = 8 at 72 h postop.; n = 5 at 7 days postop.

abbreviations: arb. – arbitrary

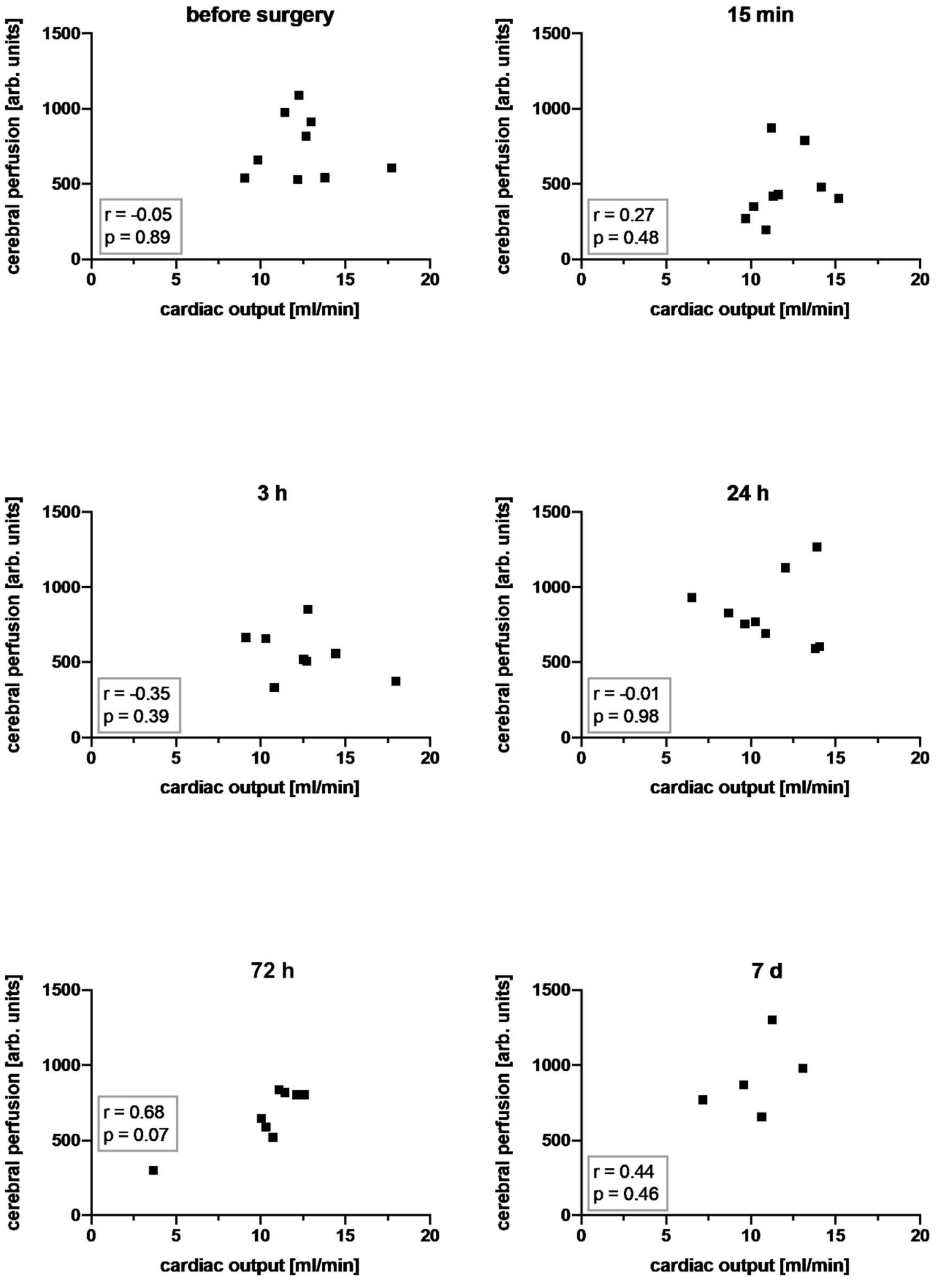

Supplemental Figure E2

Correlation of cardiac output with cerebral perfusion at the indicated times

Correlation strength, expressed as Pearson's or Spearman's correlation coefficient (r) as appropriate, with $p < 0.05$ considered statistically significant.

n = 9 for the time points before surgery and 15 min, 3 h, 24 h postop.; n = 8 at 72 h postop.; n = 5 at 7 days postop.

abbreviations: arb. – arbitrary

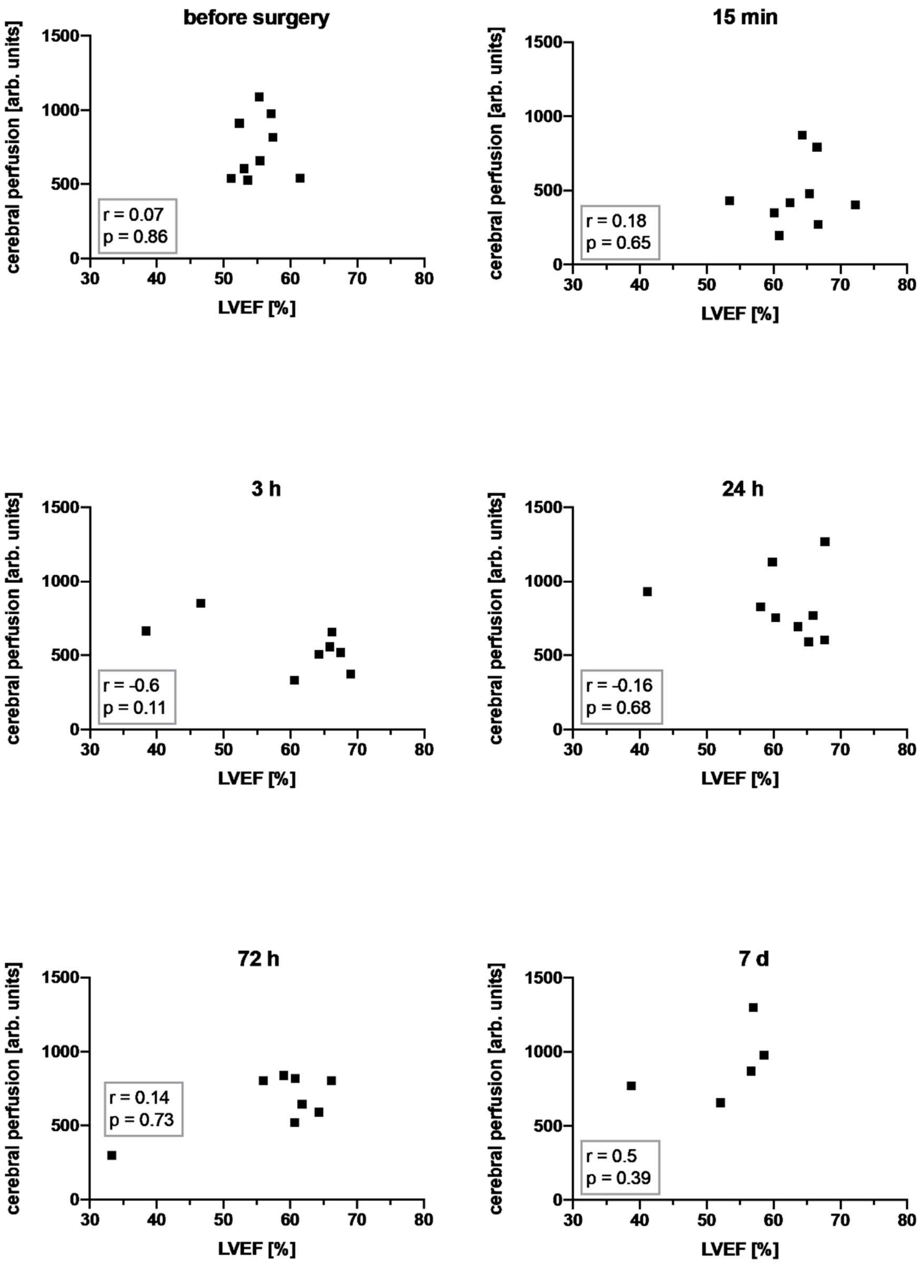

Supplemental Figure E3

Correlation of left ventricular ejection fraction (LVEF) with cerebral perfusion at the indicated times

Correlation strength, expressed as Pearson's or Spearman's correlation coefficient (r) as appropriate, with $p < 0.05$ considered statistically significant.

n = 9 for the time points before surgery and 15 min, 3 h, 24 h postop.; n = 8 at 72 h postop.; n = 5 at 7 days postop.

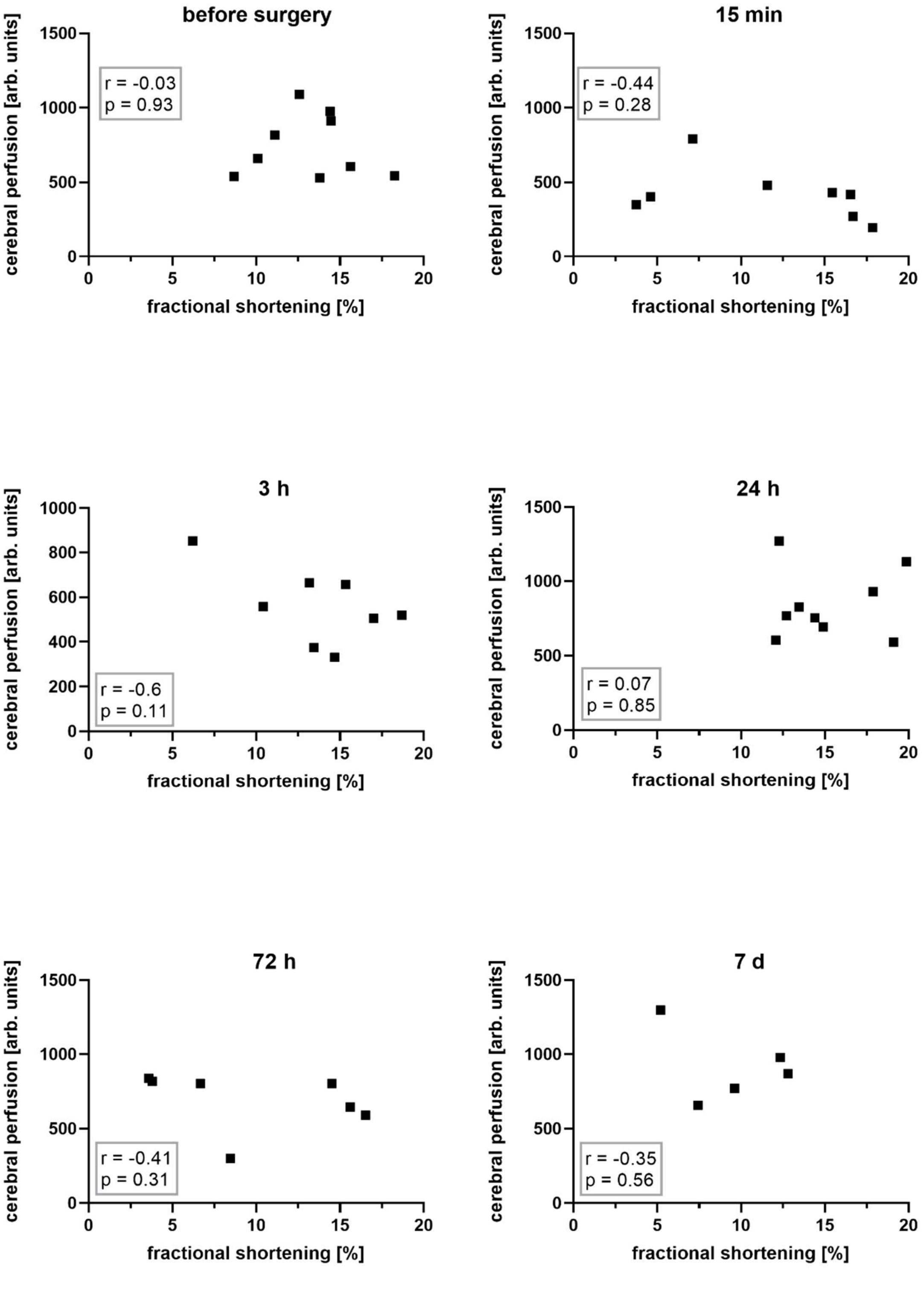

Supplemental Figure E4

Correlation of fractional shortening with cerebral perfusion at the indicated times

Correlation strength, expressed as Pearson's or Spearman's correlation coefficient (r) as appropriate, with $p < 0.05$ considered statistically significant.

n = 9 for the time points before surgery and 15 min, 3 h, 24 h postop.; n = 8 at 72 h postop.; n = 5 at 7 days postop.

abbreviations: arb. – arbitrary

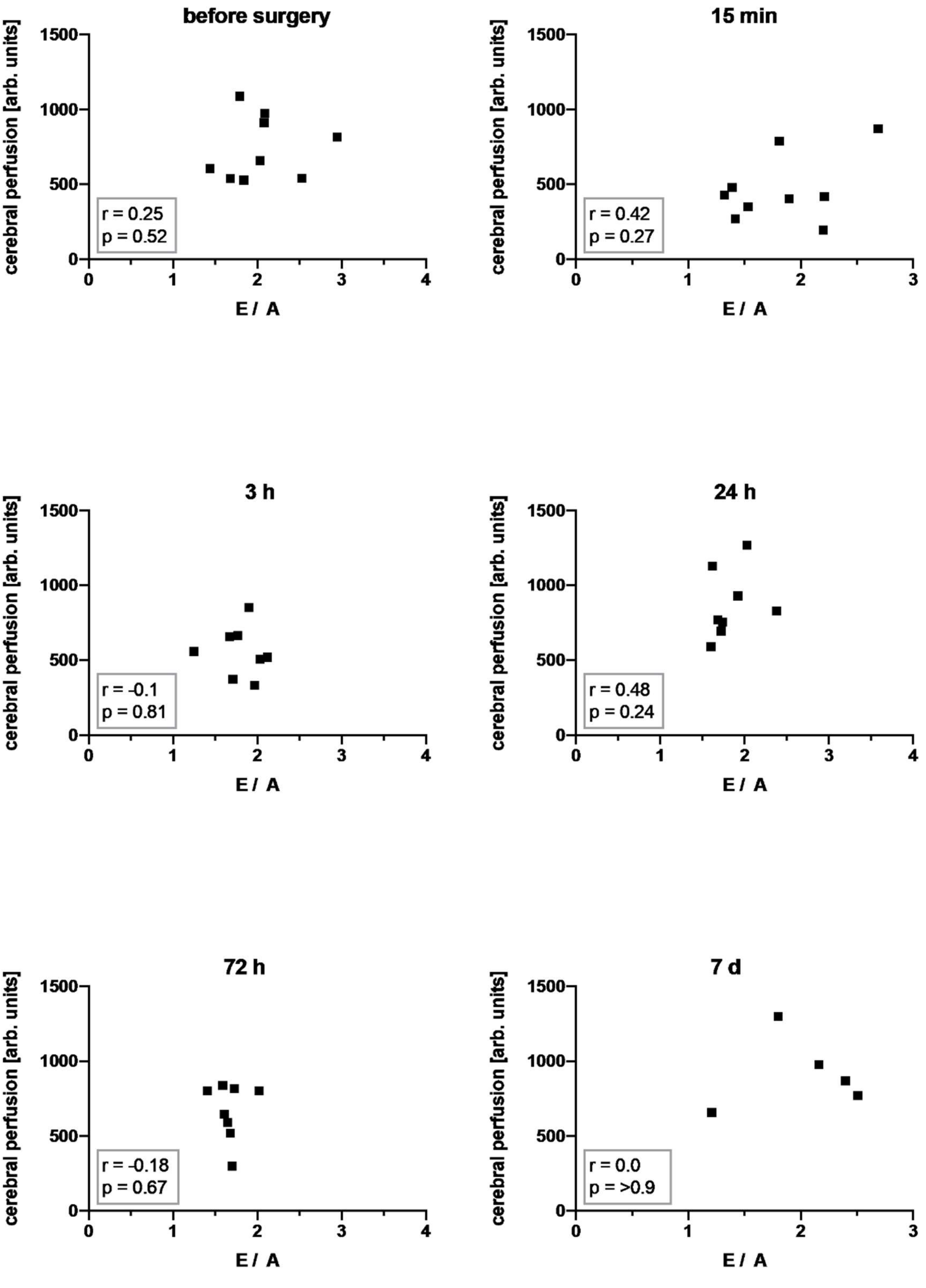

Supplemental Figure E5

Correlation of E / A with cerebral perfusion at the indicated times

Correlation strength, expressed as Pearson's or Spearman's correlation coefficient (r) as appropriate, with $p < 0.05$ considered statistically significant.

n = 9 for the time points before surgery and 15 min, 3 h, 24 h postop.; n = 8 at 72 h postop.; n = 5 at 7 days postop.

abbreviations: arb. – arbitrary

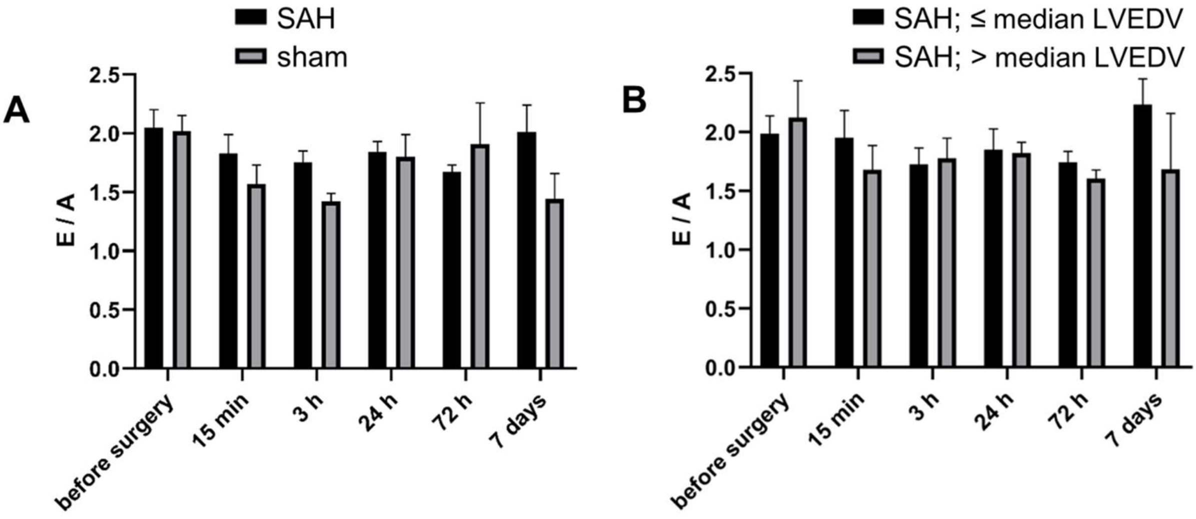

Supplemental Figure E6

Comparison of E / A in SAH and sham animals

(A) E / A values in all SAH and sham animals.

(B) E / A in SAH animals with high and low LVEDV. For each time point, the data were sorted according to median LVEDV to form two groups, one with LVEDV ≤ median and one with LVEDV > median, to test whether diastolic dysfunction contributes to low LVEDV. Note that there were no significant differences between these two groups.

The animal numbers are as follows:

(A) n = 9 SAH and n = 6 sham mice for the time points before surgery, 15 min post-surgery, and 3 h post-surgery. n = 9 SAH and n = 4 sham mice at 24 h post-surgery; n = 8 SAH and n = 4 sham at 72 h post-surgery; n = 5 SAH and n = 3 sham at 7 days post-surgery

(B) ≤ median LVEDV: n=5 for the time points before SAH and 15 min, 3 h, 24 h post-SAH; n=4 at 72 h and n=3 at 7 days post-SAH

> median LVEDV: n=4 for the time points before SAH and 15 min, 3 h, 24 h, and 72 h post-SAH; n = 2 at 7 days post-SAH

Data are presented as mean ± SEM

abbreviations: LVEDV – left ventricular enddiastolic volume; SEM standard error of the mean
